# Methodological Development of a Multi-Readout Assay for the Assessment of Antiviral Drugs against SARS-CoV-2

**DOI:** 10.3390/pathogens10091076

**Published:** 2021-08-25

**Authors:** Friedrich Hahn, Sigrun Häge, Alexandra Herrmann, Christina Wangen, Jintawee Kicuntod, Doris Jungnickl, Julia Tillmanns, Regina Müller, Kirsten Fraedrich, Klaus Überla, Hella Kohlhof, Armin Ensser, Manfred Marschall

**Affiliations:** 1 Institute for Clinical and Molecular Virology, Friedrich-Alexander University of Erlangen-Nürnberg (FAU), 91054 Erlangen, Germany; friedrich.hahn@uk-erlangen.de (F.H.); sigrun.haege@fau.de (S.H.); alexandra.herrmann@uk-erlangen.de (A.H.); christina.wangen@uk-erlangen.de (C.W.); jintawee.kicuntod@extern.uk-erlangen.de (J.K.); doris.jungnickl@uk-erlangen.de (D.J.); jul.tillmanns@fau.de (J.T.); mueller.regina@uk-erlangen.de (R.M.); kirsten.fraedrich@uk-erlangen.de (K.F.); klaus.ueberla@fau.de (K.Ü.); armin.ensser@fau.de (A.E.); 2 Immunic AG, 82166 Gräfelfing, Germany; hella.kohlhof@imux.com

**Keywords:** COVID-19 pandemic, SARS-CoV-2 infection, cultured human cells, methodological development, multi-readout assay (MRA), antiviral drug assessment, candidate drugs, anti-coronaviral treatment

## Abstract

Currently, human infections with the severe acute respiratory syndrome coronavirus type 2 (SARS-CoV-2) are accelerating the ongoing spread of the pandemic. Several innovative types of vaccines have already been developed, whereas effective options of antiviral treatments still await a scientific implementation. The development of novel anti-SARS-CoV-2 drug candidates demands skillful strategies and analysis systems. Promising results have been achieved with first generation direct-acting antivirals targeting the viral polymerase RdRp or the protease 3CL^pro^. Such recently approved or investigational drugs like remdesivir and GC376 represent a basis for further development and optimization. Here, we establish a multi-readout assay (MRA) system that enables the antiviral assessment and mechanistic characterization of novel test compounds, drug repurposing and combination treatments. Our SARS-CoV-2-specific MRA combines the quantitative measurement of several parameters of virus infection, such as the intracellular production of proteins and genomes, enzymatic activities and virion release, as well as the use of reporter systems. In this regard, the antiviral efficacy of remdesivir and GC376 has been investigated in human Caco-2 cells. The readouts included the use of spike- and double-strand RNA-specific monoclonal antibodies for in-cell fluorescence imaging, a newly generated recombinant SARS-CoV-2 reporter virus d6YFP, the novel 3CL^pro^-based FRET CFP::YFP and the previously reported FlipGFP reporter assays, as well as viral genome-specific RT-qPCR. The data produced by our MRA confirm the high antiviral potency of these two drugs in vitro. Combined, this MRA approach may be applied for broader analyses of SARS-CoV-2-specific antivirals, including compound screenings and the characterization of selected drug candidates.

## 1. Introduction

Human infection with the severe acute respiratory syndrome coronavirus type 2 (SARS-CoV-2) caused coronavirus disease 2019 (COVID-19), which was declared as a pandemic by the World Health Organization on 11 March 2020. The COVID-19 pandemic has spread rapidly since then, and today more than 210 million infections and >4.4 million deaths have been confirmed, with an estimated mortality risk of ≤2.1% [1]. Although very powerful vaccines have been produced within a short period, the spectrum of approved and effective antiviral drugs for the prevention and treatment of disease is still missing [2,3]. Remdesivir (RDV) was first approved as a direct-acting antiviral drug against COVID-19, yet its actual clinical efficacy and benefit for patients is still a matter of controversial debate [4,5].

New types of antiviral interventions, based on the generation of improved drug candidates, will likely require several years to develop. Given the urgency of the ongoing COVID-19 pandemic however, specific analysis tools and screening systems are promptly needed. Notably in this regard, SARS-CoV-2 is characterized by its unusual complexity amongst RNA viruses, not only concerning its virion structure and genomic coding capacity, but specifically the variety of regulatory proteins and viral enzymes expressed in infected cells. The nonstructural proteins, including a 3-chymotrypsin-like protease (3CL^pro^), papain-like protease, helicase, RNA-dependent RNA polymerase (RdRp), and several more, have been recognized as attractive targets for developing antiviral drugs as well as options for drug combination and repurposing strategies [6,7,8,9]. Initial analyses point to the promising potential of the prototype inhibitors of RdRp (RDV, favipiravir, ribavirin and others [1]) as well as 3CL^pro^ (GC376, boceprevir, ivermectin, tipranavir and others; [1,10,11]).

The further development of these anti-SARS-CoV-2 strategies and the profound in vitro characterization of the experimental hits and developmental candidates is greatly dependent on the multidimensional nature and quality of test systems. These may either represent relatively simple measurements, such as viral enzymatic in vitro assays or standard cultured-cell infection tools, to be performed on large screening scales with an advantageous ease of application. Or these may represent more complex systems, offering the ability to assess antiviral drug effects, including a determination of their mode-of-action (MoA), an in vitro characterization in physiologically appropriate cell types, drug resistance profiling or proof-of-concept (PoC) studies. In this report, we describe a multi-readout system (MRA) for the measurement of the inhibitory characteristics of anti-SARS-CoV-2 compounds in a disease-relevant human cell line. This MRA system combines the quantitative and qualitative measurements of several parameters of virus infection. With the study, we intended to emphasize the high usefulness of this system for SARS-CoV-2 research that can be applied for comparative analyses of specific viral strains and may be rapidly adapted to newly emerging variants. Putative options of a current use in the ongoing COVID-19 antiviral drug research are discussed.

## 2. Results

### 2.1. Establishment of the SARS-CoV-2 Infection System in Human Caco-2 Cells for Isolate MUC-IMB-1/2020 and SARS-CoV-2 Reporter Virus

**Figure 1:**
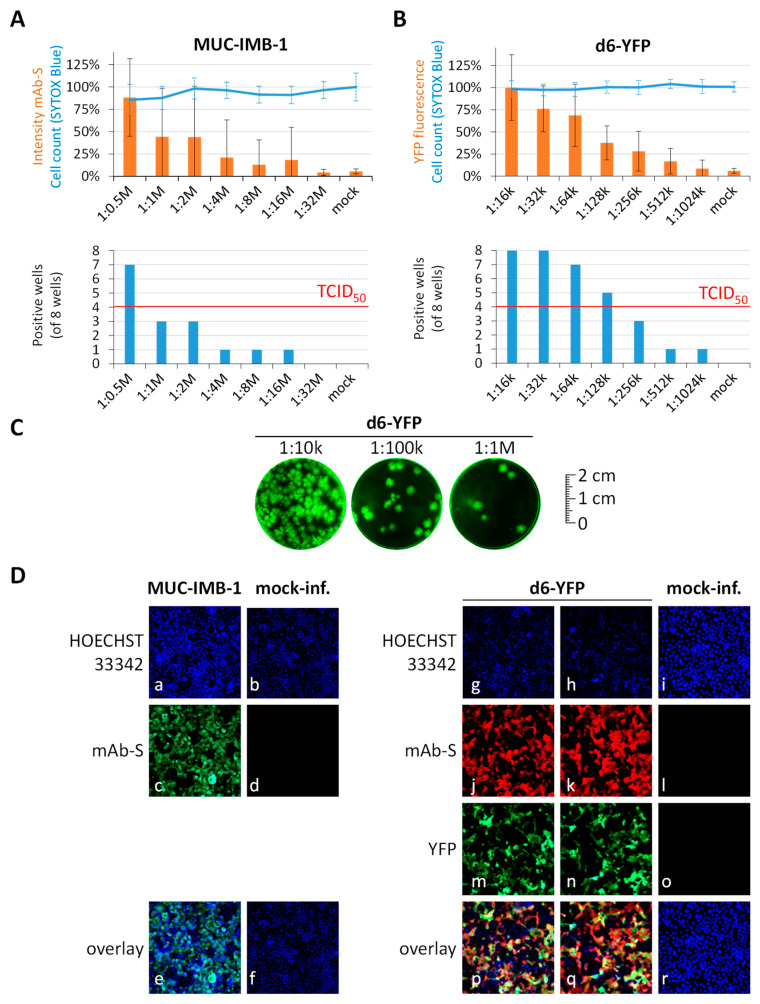
Titration experiments of SARS-CoV-2 stock viruses. (**A**) MUC-IMB-1 isolate determined by immunostaining using mAb-S versus cell viability indicated by SYTOX Blue staining. (**B**) Reporter virus d6-YFP determined by reporter signal quantities using YFP fluorescence versus cell viability indicated by SYTOX Blue staining. Caco-2 cells were cultivated in 96-well plates (25,000 cells/well), infected with serial dilutions of the stock viruses as indicated, and harvested for evaluation at 48 h post-infection (h p.i.). Measurements were performed in the 96-well format as octuplicate determinations (values are given as mean ± SD, and one representative experiment is shown). Positive wells were defined as fluorometry-positive when reaching significantly higher background signals. The x-axes defined dilutions (1:0.5 M to 1:32 M or 1:16 k to 1:1024 k, respectively) of viral inoculi. (**C**) Plaque formation assay of d6-YFP. Caco-2 cells infected with indicated dilutions of the d6-YFP reporter virus were treated with an Avicel overlay to limit the spread of infection. Viral plaques were detected 3 d p.i. by fluorescence imaging. (**D**) Images of the two viruses were recorded with an ImmunoSpot Image Analyzer for the signals of viral spike protein expression (mAb-S), reporter expression (YFP), cell viability (HOECHST 33342), and an overlay of the respective signals is given (scale bars could not be provided as a limitation of this technical device). M, million; k, thousand.

In a first step of the analysis, viral stocks were produced in Caco-2 cells and their application in quantitative antiviral assays was prepared by endpoint titration experiments. For both the clinical isolate of SARS-CoV-2, MUC-IMB-1/2020 (Figure 1A), and the SARS-CoV-2 reporter virus, termed d6-YFP (Figure 1B), serial dilutions of the stocks were used for the infection of Caco-2 cells. Viral titers were determined by the readout of an in-cell immunofluorescence assay (mAb-S) or a reporter-based fluorescence measurement (YFP), respectively. Moreover, the viral titer of d6-YFP determined by endpoint titration was confirmed in a plaque formation assay by directly utilizing the quantitative signals of virus-induced fluorescence of the infected cell plaques (Figure 1C).

**Figure 2:**
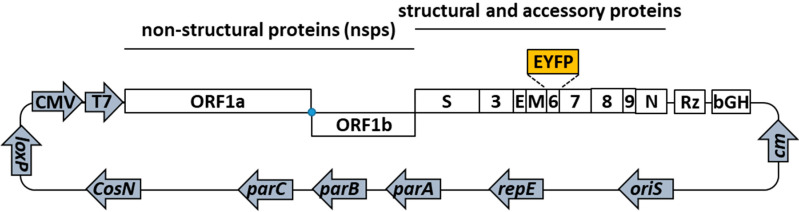
Schematic depiction of pBSCoV2 d6-YFP as the genetic basis to reconstitute the novel rec-SARS-CoV-2 d6-YFP virus. The BACmid pBSCoV2 d6-YFP encodes a full-length SARS-CoV-2 genome, in which the open reading frame (ORF) 6 was replaced by the enhanced yellow fluorescent protein (EYFP) through homologous recombination. Non-structural proteins (nsp) 1 to 16 are encoded within the ORFs 1a and 1b, which are translated by ribosomal frameshifting (blue dot) during cap-dependent translation. Structural and accessory proteins are translated by their respective sub-genomic RNAs and include spike (S), ORF3, envelope (E), matrix (M), ORF6 to ORF9, and the nucleoprotein (N). The genome of SARS-CoV-2 was assembled into a modified pBeloBAC11 backbone containing the human cytomegalovirus (CMV) immediate early gene (IE) promoter-enhancer and T7 RNA polymerase (T7) promoter, the hepatitis delta virus ribozyme (Rz) as well as the bovine growth hormone (bGH) polyadenylation signal. Cm, chloramphenicol resistance; oriS, origin of viral replication; repE, replication initiation factor of *E. coli*; parA, ATPase; parB, DNA-binding protein; parC, cis-acting sequence (parA/B/C required for successful partitioning of low copy plasmids); CosN, site for packaging into lambda phage particles; loxP, target site for specific cleavage by Cre recombinase.

The d6-YFP reporter virus was newly generated by two-step Red recombination. In brief, an anonymized patient sample of SARS-CoV-2 was used as a template for RNA-specific RT-PCR genome amplification (Figure 2). Four resulting amplicons were assembled with a modified pBeloBAC11 backbone (ref [12] pBeloBAC11, GenBank accession U51113, New England Biolabs/Addgene), containing CMV and T7 promotors, the HDV ribozyme as well as a bGH polyA signal. Therein, the viral open reading frame 6 gene (ORF6) was replaced by an enhanced yellow fluorescent protein (EYFP) expression module by two-step homologous recombination [13]. Virus reconstitution was achieved by transfecting a co-culture of two 293T lines, either expressing human ACE2 or the viral N protein to produce rec-SARS-CoV-2 d6-YFP (d6-YFP) which was further propagated in Caco-2 cells. In the titration settings of stock viruses, the dilutions of infectious inoculi, to be used in the quantifiable range of Caco-2 infection, were thereby ascertained for the chosen conditions as given by the half-maximal tissue-culture infectious dose (TCID_50_). A visualization of the degree of infection at 1 × TCID_50_ is presented by microscopic images (Figure 1D, see panels a-f for MUC-IMB-1 and g-r for d6-YFP, note the mAb-S signals in c-d compared to mAb-S plus YFP signals in j-o, respectively). Thus, both viral strains were found to be suitable for the use on a broader scale of methodological development in the Caco-2 cell system.

### 2.2. Multiple Readouts of Measuring Antiviral Drug Activity for Clinically Relevant and Reporter-Based SARS-CoV-2 Viruses

**Figure 3:**
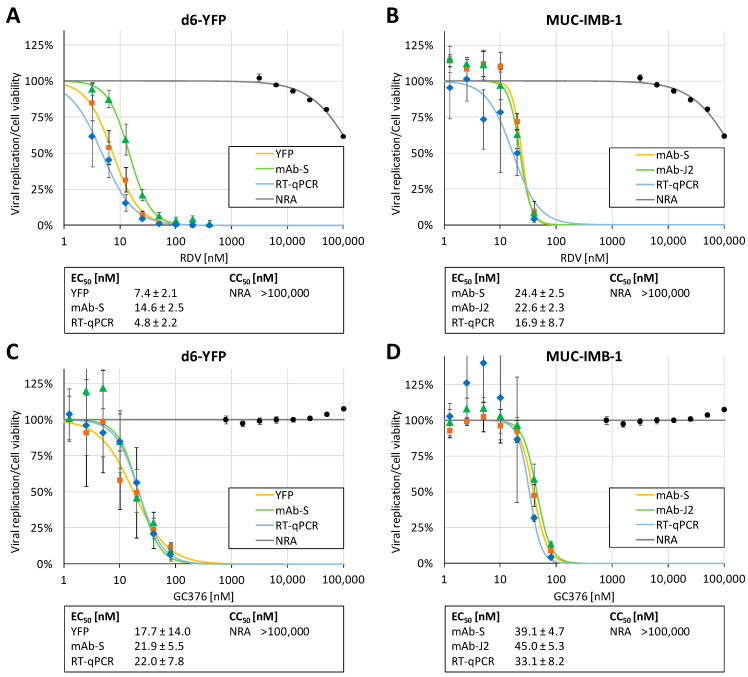
Comparative analysis of infection with the two SARS-CoV-2 viruses MUC-IMB-1 and d6-YFP in Caco-2 cells, using multiple readouts as indicated. Antiviral activity of the two drugs RDV (**A**,**B**) and GC376 (**C**,**D**) was assessed in parallel. Caco-2 cells were cultivated in 96-well plates at 25,000 cells/well, infected with SARS-CoV-2 d6-YFP (**A**,**C**) or MUC-IMB-1/2020 (**B**,**D**) at an MOI of 0.003 and harvested at 30 h p.i. Viral replication was determined by RT-qPCR of viral genomes in the cell culture supernatants as well as multiple combinations of quantitative fluorescent detections using the fixed cells, including viral YFP expression and antibody-mediated detection of the indicated viral antigens. Three independent biological replicates were performed for both viruses MUC-IMB-1 and d6-YFP, using the different readouts, and one representative experiment is shown. Measurements were performed in the 96-well format as quadruplicate determinations (values are given as mean ± SD). Cell viability was determined from parallel cultures of uninfected Caco-2 cells drug-treated for 48 h, and is presented as mean values of triplicate determinations ± SD.

Next, SARS-CoV-2 infection assays were performed to assess the antiviral activity of two reference drugs in the Caco-2 system and to establish multiple readouts for future screenings and further applications of antiviral drug analysis. RDV was the first FDA-approved drug for SARS-CoV-2 treatment, acting as a nucleoside analog prodrug. GC376 represented an investigational drug candidate, acting as an inhibitor of the viral main protease 3CL^pro^. Here, we applied antiviral measurements for these two direct-acting antivirals (DAAs), RDV (Figure 3A,B) and GC376 (Figure 3C,D), using the two viral strains as a new approach to compare clinically relevant and reporter-based SARS-CoV-2 viruses under comparable conditions of in vitro settings of infection. For d6-YFP infections, the applied readouts were YFP reporter fluorometry, the in-cell immunofluorescence assay using the mAb-S antibody, and viral genome-specific RT-qPCR; for MUC-IMB-1/2020 the readouts were the in-cell immunofluorescence assay (using both the mAb-S and the mAb-J2 double-strand RNA [dsRNA]-specific antibodies) and RT-qPCR. Neutral Red assay (NRA) was performed to dissect antiviral activities from the ranges of cytotoxicity (Figure 3A–D, CC_50_ values > 100,000 nM for RDV as well as GC376). Importantly, the EC_50_ values for the individual readouts indicated that the inhibitory potential of both drugs was within a relatively consistent range of data, and thus confirmed the reliability of all measurements. For RDV, referring to either of the two viruses, the EC_50_ values of the readouts were (A) 7.4 ± 2.1, 14.6 ± 2.5, 4.8 ± 2.2 nM and (B) 24.4 ± 2.5, 22.6 ± 2.3, 16.9 ± 8.7 nM; for GC376, the EC_50_ values were (C) 17.7 ± 14.0, 21.9 ± 5.5, 22.0 ± 7.8 nM and (D) 39.1 ± 4.7, 45.0 ± 5.3, 33.1 ± 8.2 nM. This indicated that, within a given range of biological variability, the readouts of this MRA were consistent and the comparative analysis underlined the strong antiviral in vitro efficacy of these drugs. Specifically, the parameters of viral replication addressed by the individual readouts conclusively showed the drug-mediated inhibition of SARS-CoV-2 replication in Caco-2 cells at several replicative stages, such as the reporter expression, block of intracellular production of viral proteins or RNA, virion release and viral genomic load. It should also be emphasized that the protocol based on the d6-YFP reporter virus greatly eases handling and improves capacities with higher sample numbers compared to the non-reporter parental virus. Thus, the MRA may facilitate a number of applications in anti-SARS-CoV-2 drug research.

### 2.3. Use of Two Plasmid-Based Assay Systems to Screen for Novel Viral 3CL^pro^ Protease Inhibitors under Noninfectious Conditions

#### 2.3.1. The FRET CFP::YFP-Based 3CL^pro^ Activity Assay (FRET Assay)

**Figure 4:**
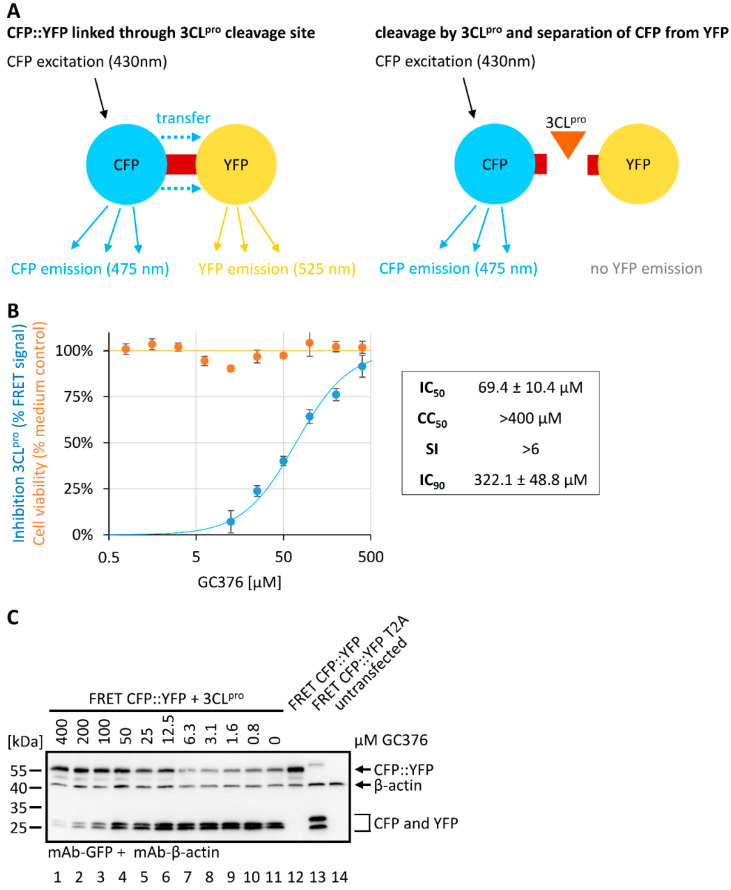
Establishment of the new FRET CFP::YFP-based 3CL^pro^ activity assay. (**A**) The principle of this assay system is based on the Förster resonance energy transfer (FRET) from CFP to YFP. The fusion of the CFP::YFP construct is linked by a 3CL^pro^-sensitive cleavage site and thus the intrinsic FRET signal can be disrupted through the activity of 3CL^pro^ by separating the CFP from the YFP moiety. (**B**) The 293T cells were used for transient transfection with the reporter pair FRET CFP::YFP and 3CL^pro^ or the controls FRET CFP::YFP (FRET-positive) or self-cleaving FRET CFP::YFP-T2A (FRET-negative). At 4 h post-tranfection (h p.t.), cells were treated with GC376 at indicated concentrations. At 19 h p.t., the FRET signal was measured by a Victor X4 microplate reader. Two independent biological replicates were performed and one representative experiment is shown. Measurements were performed in the 96-well format as triplicate determinations, values are given as mean ± SD. Inhibitory activity of the GC376 was assessed and set in relation to cell viability (Neutral Red assay) as indicated. (**C**) Western blot control stainings with mAb-GFP and mAb-β-actin were performed as depicted.

In our attempts to utilize plasmid-based assay systems for analyzing the inhibitors of the 3CL^pro^ protease, we first established a novel approach on the basis of cyan and yellow fluorescent proteins (CFP, YFP) in a CFP::YFP fusion construct. The fusion of CFP and YFP allowed for an energy transfer (FRET) from the excited CFP to the fused YFP leading to a measurable YFP emission. This so-called intrinsic FRET signal could be disrupted by the separation of the two fluorescent moieties, achieved in our construct by the activity of the 3CL^pro^. To this end, a transient expression plasmid for the FRET CFP::YFP fusion was generated in our laboratory, in which the two fluorescent moieties were linked through a 3CL^pro^-specific cleavage site, and used for cotransfection experiments together with the 3CL^pro^-encoding plasmid. The principle of this FRET assay was based on the idea that any addition of a 3CL^pro^-directed inhibitor, here represented by the reference compound GC376, blocked the cleavage of CFP::YFP and thus maintained the FRET signal (Figure 4A).

**Figure 5:**
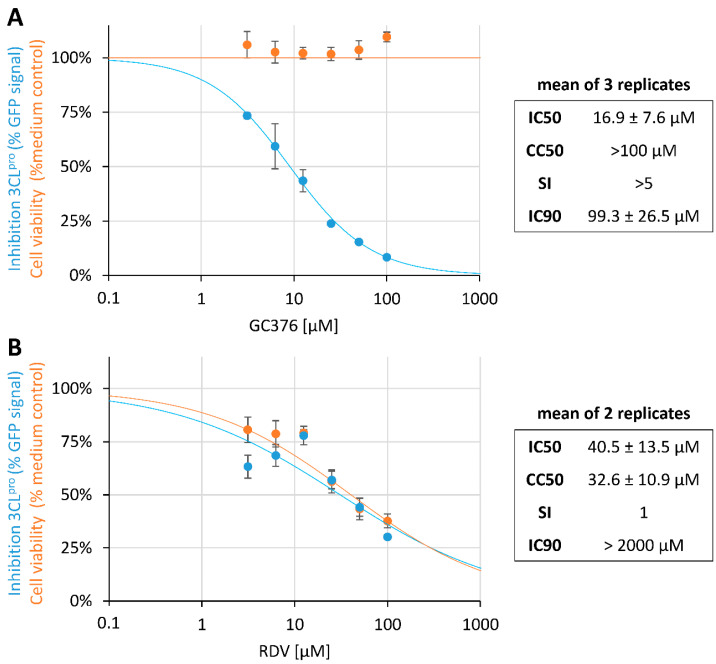
The FlipGFP-based 3CL^pro^ activity assay [16] was applied to determine protease-directed inhibitory drug activity in vitro. The 293T cells were used for transient transfection with the reporter pair FlipGFP and 3CL^pro^. At 4 h p.t., cells were treated with GC376 at indicated concentrations. At 19 h p.t. the GFP signal was measured by a Victor X4 microplate reader. Inhibitory activity of the two reference compounds GC376 (**A**) and RDV (**B**) was assessed as indicated and set in relation to cell viability (Neutral Red assay). One representative experiment out of three replicates (GC376) or two replicates (RDV) is shown and measurements were performed in the 96-well format as triplicate determinations, values are given as mean ± SD.

Although the approach of using FRET-based reporter systems was not new and had been applied in various contexts, we adapted the FRET tool to 3CL^pro^-specific use in analyzing protease candidate inhibitors. The measurement of a series of increasing GC376 concentrations incubated on the reporter/3CL^pro^-cotransfected cells indeed confirmed the drug-mediated restoration of the FRET signal, corresponding to a 3CL^pro^-specific IC_50_ value of 69.4 ± 10.4 µM in this system. It should be emphasized that the levels of inhibitory IC_50_ values strongly correlated with the individual cell-based 3CL^pro^ test systems (compare Figure 5). These systems represented cellular enzyme-based tools in an overexpression situation that allowed for a quantitative assessment, but did not represent physiological target protein levels. For this reason, no simple transfer of the IC_50_ values determined in the 3CL^pro^-specific reporter systems was possible toward the EC_50_ values of the SARS-CoV-2 infection systems (compare Figure 3). Thus, each of these reporter systems should be regarded as an individual unit of drug assessment, so that only a relative comparison between reference drugs and screening hits or new candidate compounds allowed the statement on drug efficacies. As another readout in this system, Western blot analysis was performed using aliquots of the total FRET assay lysates in order to verify the 3CL^pro^-specific cleavage of the fusion construct (Figure 4C). FRET CFP::YFP T2A was a self-cleaving control construct that contained the T2A peptide [14] instead of the 3CL^pro^ cleavage site (Figure 4C, lane 13). The inhibition of cleavage products CFP and YFP was detected in a GC376 concentration-dependent manner (Figure 4C, lanes 1–11). Thus, the FRET CFP::YFP-based 3CL^pro^ activity assay was considered as a new option of in vitro compound testing and primary screening.

#### 2.3.2. The FlipGFP-Based 3CL^pro^ Activity Assay (FlipGFP Assay)

Next, we used the recently developed FlipGFP-based 3CL^pro^ activity assay [15,16,17]. This plasmid-encoded reporter assay was based on the expression of a conformation-specific version of the green fluorescent protein (GFP), which emitted fluorescence only after cleavage by the viral 3CL^pro^ protease, as coexpressed from a separate plasmid. According to the experimental optimization of the specific conditions of analysis, the FlipGFP assay allowed for an initial 3CL^pro^-directed antiviral drug screening in the absence of SARS-CoV-2 infection. Here, we optimized the protocol by utilizing the reference drug GC376. In particular, the ratio between plasmid concentrations for the expression of 3CL^pro^ and FlipGFP had been serially varied in a scale of comparative settings (Figure S1), so that optimal ratios of 3CL^pro^/FlipGFP in the range of 0.6 + 1 to 0.2 + 1 could be experimentally defined. Using these specific conditions, the IC_50_ and IC_90_ values of GC376 were determined with 16.9 ± 7.6 µM and 99.3 ± 26.5 µM, respectively (Figure 5A), in the absence of drug-induced cytotoxicity (CC_50_ >100 µM, selectivity index SI > 5). As a control, the mechanistically different anti-SARS-CoV-2 drug RDV was used in parallel and, as expected, did not show a 3CL^pro^ inhibitory activity (Figure 5B; SI 1, note that the IC_50_ and CC_50_ values of RDV lie within a very narrow range and an extrapolated IC_90_ > 2000 µM). This approach confirmed previous data on the anti-SARS-CoV-2 potential of GC376 [10,11], and supported the reliability of this reporter assay for comprehensive studies of 3CL^pro^-directed drugs.

### 2.4. Combinatorial Drug Assessment Using the SARS-CoV-2 d6-YFP Reporter System

**Figure 6:**
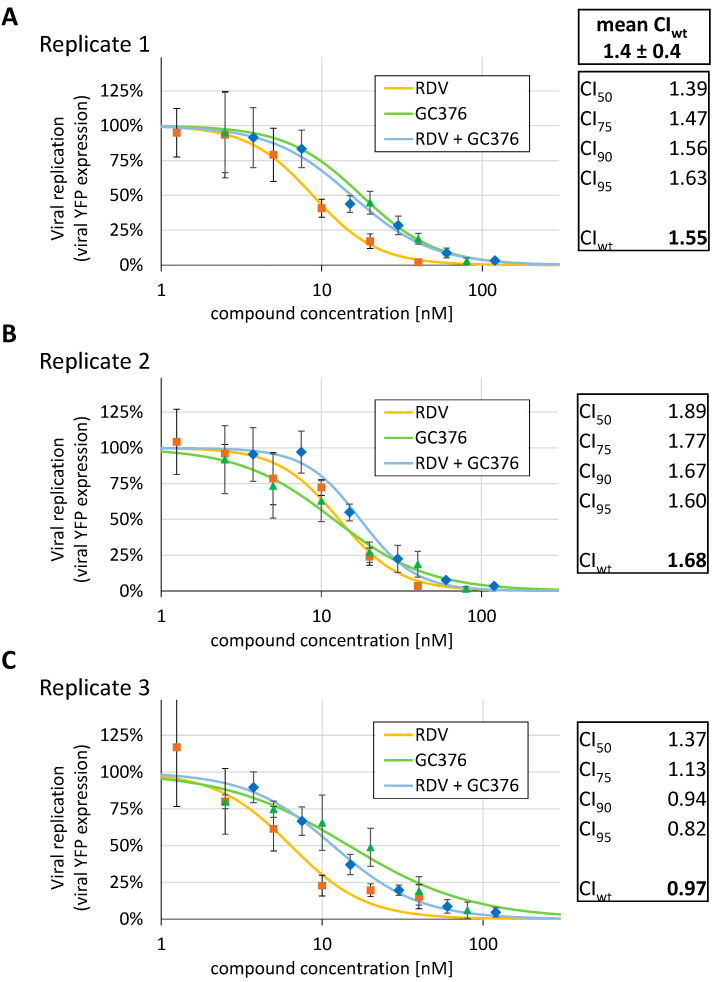
Combinatorial drug assessment of RDV and GC376 using the rec-SARS-CoV-2 d6-YFP reporter system. Caco-2 cells were cultivated in 96-well plates at 25,000 cells/well, infected with SARS-CoV-2 d6-YFP at an MOI of 0.003 and treated with RDV, GC376 or a combination of the drugs, starting at the respective 4 × EC_50_ concentrations of the single compounds. Viral replication was determined as 30 h p.i. by quantitative fluorescence detection of virus-driven YFP expression in the fixed cells. Inhibitory profiles of viral replication measured through virus-encoded YFP reporter expression for Replicate 1 (**A**), Replicate 2 (**B**) and Replicate 3 (**C**) are presented as dose reponse plots for RDV, GC376 and the combination RDV + GC376. Three independent biological replicates were performed as shown, and measurements were performed in the 96-well format as quadruplicate determinations, values are given as mean ± SD. The combinatorial drug assessment was calculated by using the CompuSyn algorithm. CI values at 50, 75, 90 and 95% virus inhibition were used for the final evaluation.

Additionally, the combinatorial drug treatment of SARS-CoV-2 infection was analyzed using the d6-YFP reporter system. To this end, the Loewe additivity fixed-dose assay was performed under conditions applied from our previous studies [18]. Essentially, the cotreatment with RDV plus GC376 was expected to have a non-antagonistic, possibly additive or synergistic effect, considering the fact that the two drugs acted in mechanistically different modes of action (MoA), i.e., a nsp12 replication complex-directed MoA by nucleoside analog RDV, and a 3CL^pro^-directed MoA by the protease inhibitor GC376. The results obtained from three experimental replicates actually indicated that no synergism was detectable upon the cotreatment with RDV and GC376 (Figure 6A–C, Replicates 1–3). The CompuSyn algorithm-based analysis of the data asserted an additive effect of this combination treatment (Figure 6A, mean CI_wt_ of 1.4). However, part of these data also hinted at antagonistic interference (in particular CI_wt_ of 1.68 in Replicate 2, Figure 6B), as there seemed to be no perfect separation between the MoAs of these two drugs, at least in vitro. The latter may be an in vitro effect only arising under the chosen experimental conditions. It may likewise be based on a negative drug interference in terms of cellular uptake or stability, or, alternatively, on secondary drug targeting effects. Nevertheless, given the complexity of coronaviral gene regulation and genome replication, this question should be taken into closer consideration before addressing this type of combination treatment in vivo.

### 2.5. Analysis of the Anti-SARS-CoV-2 Activity of CDK Inhibitors Determined by Multiple Readouts

**Figure 7:**
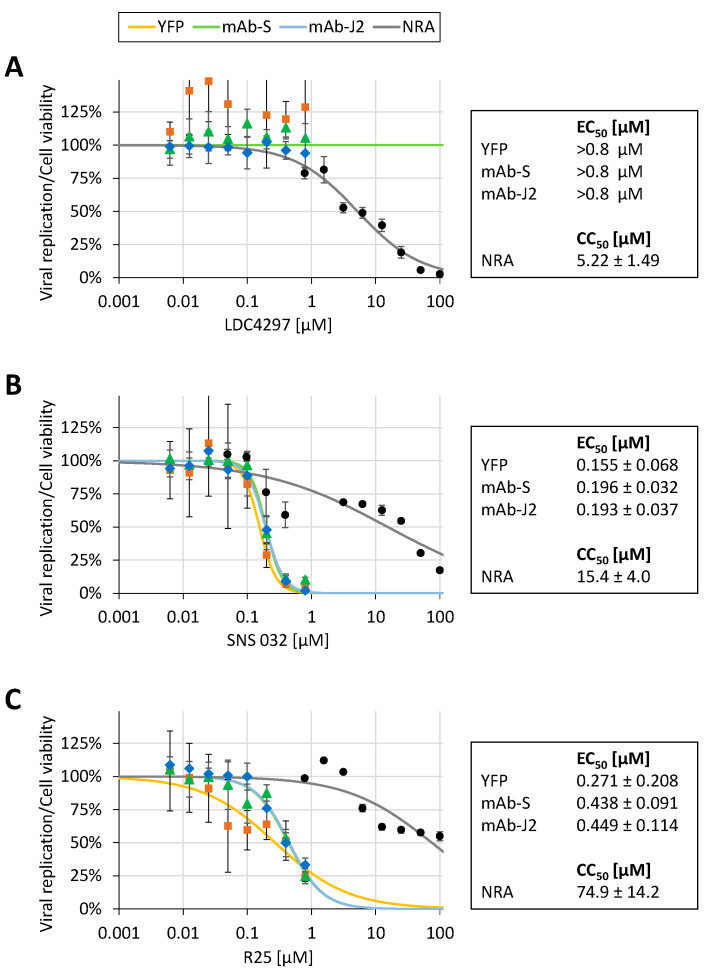
Antiviral activity of selected CDK inhibitors in SARS-CoV-2 d6-YFP-infected Caco-2 cells by using multiple readouts. Caco-2 cells were cultivated in 96-well plates at 25,000 cells/well, infected with SARS-CoV-2 d6-YFP at an MOI of 0.003 and treated with the indicated concentrations of LDC4297 (**A**), SNS 032 (**B**), or R25/alsterpaullone (**C**). At 30 h p.i., cells were harvested and viral replication was determined by multiple combinations of quantitative fluorescence detections using the fixed cells, e.g., viral YFP expression and antibody-mediated detection of spike protein (mAb-S) or dsRNA (mAb-J2). Measurements were performed in the 96-well format as quadruplicate determinations, values were given as mean ± SD. Cell viability was determined from parallel cultures of uninfected Caco-2 cells drug-treated for 48 h (presented as mean values of triplicate determinations ± SD). Up to three independent biological replicates were performed for these three CDK inhibitors and one representative experiment is shown.

In order to investigate the potential of host-directed antivirals (HDAs), as a second example of the application of this MRA system, we analyzed three inhibitors of cellular cyclin-dependent kinases (CDKs). These CDK inhibitors, i.e., SNS 032, R25/alsterpaullone and LDC4297 that were directed against CDK9, CDK1/2/5 or CDK7, respectively, had previously been characterized for their antiviral properties by using a number of different virus systems [18,19,20,21,22,23]. Despite the broad in vitro and in vivo antiviral activities reported for LDC4297, which included human and animal herpesviruses, vaccinia virus, human adenovirus type 2, human immunodeficiency virus type 1 and influenza A virus [19], in the present analysis LDC4297 did not inhibit SARS-CoV-2 replication at non-cytotoxic concentrations (Figure 7A). However, in contrast to LDC4297, the CDK9 inhibitor SNS 032 potently inhibited SARS-CoV-2 replication with EC_50_ values of 0.155 ± 0.068, 0.196 ± 0.032 and 0.193 ± 0.037 µM when using YFP, mAb-S or mAb-J2 as the respective readouts (Figure 7B). Cell viability measurements revealed a CC_50_ value of 15.4 µM resulting in an SI from approximately 80 to 99. Similarly, the inhibition of CDK1/2/5 by R25 reduced the SARS-CoV-2 replication efficiency with EC_50_ values of 0.271 ± 0.208, 0.438 ± 0.091 and 0.449 ± 0.114 µM for the respective readouts (Figure 7C). The CC_50_ value for R25 was determined as 74.9 ± 14.2 µM resulting in an SI from approximately 167 to 276. Taken together, the MRA consistently demonstrated an independence of SARS-CoV-2 replication from CDK7 activity, whereas the inhibition of CDK1/2/5 or CDK9 exerted a pronounced anti-SARS-CoV-2 activity in vitro.

## 3. Discussion

### 3.1. Summarized Achievements in Developing a Multi-Readout Assay for the Assessment of Anti-SARS-CoV-2 Drugs

Novel options of antiviral prevention and treatment against the worldwide problem of COVID-19 are urgently needed. So far, the development of anti-SARS-CoV-2 drug candidates has been hampered by the complexity of the SARS-CoV-2 genome and genetic variability as well as the unusually complex gene regulation. Moreover, the intensified coronavirus-specific experimentation in numerous laboratories revealed the challenges of building up reliable cell culture systems for the quantitative assessment of the in vitro replication of SARS-CoV-2 reference strains, clinical isolates and rapidly evolving mutants. In this study, we focused on the methodological establishment of an MRA system that, on the one hand, offers an ease of handling and, on the other hand, enables the antiviral characterization of novel compounds at various levels, including both DAAs and HDAs, as well as drug repurposing and combination treatments.

This MRA system combined the quantitative measurement of the parameters of virus infection such as the intracellular production of proteins and genomes, enzymatic activities, virion production and release, with the generation of reporter systems and recombinant viruses. The novelty of our findings was demonstrated by several points: (i) human Caco-2 cells were infected with SARS-CoV-2 (clinical isolate or recombinant virus) and could be used for the MRA evaluation of drug sensitivity by immunofluorescence imaging with spike- and double-strand RNA-specific monoclonal antibodies, in-cell ELISA and virion release-specific RT-qPCR; (ii) the recombinant SARS-CoV-2 reporter virus d6-YFP proved to be highly reliable in antiviral measurements, and may also be used as a genetic basis for the generation of further site-specific reporter mutants; (iii) two plasmid-based reporter modules, i.e., the FlipGFP and FRET assays directed to the viral 3CL^pro^ enzymatic activity, were comparatively applied in this study; and (iv) the first data on drug combination treatment were collected for the combined in vitro administration of inhibitors directed to the RdRp and 3CL^pro^.

The focus on Caco-2 cells in this study underlined previous reports illustrating that the in vitro efficacy of antiviral compounds analyzed for SARS-CoV-2, at least in part, has shown a substantial variation dependent on the individual cell type, test conditions and readouts (ref [24] and references therein). Specifically, the EC_50_ values determined in human cells compared to non-human primate Vero cells revealed marked quantitative differences for distinct compounds, a variation that could be assigned to differences in cellular transport systems, drug uptake or metabolism [25]. In the present study, we were able to characterize the optimized conditions of the MRA system exerting a high degree of stability within the Caco-2 cell model, a basic quality that is of high importance for the continued preclinical/clinical development of antiviral drugs.

All of this considered, this study strongly supports the ongoing antiviral drug analysis in the face of the COVID-19 pandemic. Despite the success of vaccinations and hygienic measures, an effective SARS-CoV-2 antiviral treatment is urgently needed. Antiviral treatment might also reduce viral shedding and mitigate the spread of infection, but is primarily needed to limit disease progression and decrease both the morbidity and mortality of SARS-CoV-2 infections. Our methodological study, which focused on the development of diverse options of MRAs, reporter modules and the use of different viral strains, may facilitate the identification of SARS-CoV-2 inhibitory drug candidates, which can hopefully then be rapidly translated into clinical use.

### 3.2. Future Research Directions

By the use of the methodological platform described in the present report, further options for a variety of different viral strains and situations of infection may be provided with an extended series of recombinant SARS-CoV-2 reporter viruses currently generated in our laboratory (Herrmann, Ensser et al., manuscript in preparation). These additional viral reporter constructs were characterized by variable sites of different reporter insertion within the viral genome for individual purposes. Such SARS-CoV-2 molecular clones shall be further developed with the aim to facilitate antiviral drug analyses, studies of virus tropism and the development of further vaccine candidates. In particular, such engineered adaptations may be designed in response to newly emerging pathogenic SARS-CoV-2 variants of concern (VOCs). In addition, the expected challenges associated with the occurrence of antiviral drug resistance [26,27] as well as vaccine escape mutants [28,29] may be experimentally investigated via this methodology. In summary, on the basis of the technological achievements, future emerging issues of medical impact imposed by the SARS-CoV-2 pandemic may be addressed in a more efficient and focused manner.

## 4. Materials and Methods

### 4.1. Cell Culture and SARS-CoV-2 Infection

Human Caco-2 cells were cultivated at 37 °C, with 5% CO_2_ and 80% humidity using Dulbecco’s modified Eagle medium (DMEM, 11960044, Thermo Fisher Scientific, Waltham, MA, USA) supplemented with 2 mM GlutaMAX^TM^ (35050038, Thermo Fisher Scientific), 10 µg/mL gentamycin (22185.03, SERVA, Heidelberg, Germany), 10% fetal bovine serum (FBS, F7524, Sigma Aldrich, St. Louis, MO, USA) and 1% MEM Non-Essential Amino Acids Solution (11140050, Thermo Fisher Scientific). SARS-CoV-2 (MUC-IMB-1/2020, passage ER-P2-2, Bundeswehr Institute of Microbiology, Munich, Germany), initially propagated on Vero E6 cells (ATCC^®^ CRL-1586), was passaged twice in Caco-2 cells before being used for the infection of these cells. For generation of virus stocks, supernatants were harvested after 50 h and aliquots were stored at −80 °C. Viral titers were determined by endpoint titration on Caco-2 cells. For infection experiments, 25,000 Caco-2 cells were seeded in 96-well plates and inoculated with SARS-CoV-2 at an MOI of 0.003. Infection volumes of 200 µL contained antiviral test compounds or solvent control. Supernatants and cells were harvested 30 h p.i.. For further downstream analysis, cells were washed with PBS and fixed with 10% formalin at room temperature overnight. Viral supernatants were inactivated for 10 min at 95 °C in sealed plates. Viral replication was assessed by variable readouts as indicated and was presented as mean values of biological quadruplicates ± SD. All infection experiments were performed under BSL-3 conditions.

### 4.2. Cloning and Reconstitution of Rec-SARS-CoV-2 d6-YFP

An anonymized residual sample from a patient with SARS-CoV-2 infection was used as a template for genome amplification. Total nucleic acids of a respiratory swab sample were extracted on an automated Qiagen EZ1 analyzer using the Qiagen EZ1 virus mini kit v2.0 according to the manufacturer’s instructions. Genomic viral RNA was reverse transcribed using the LunaScript RT SuperMix Kit (NEB) according to the manufacturer’s protocol. Four overlapping fragments covering the entire viral genome were amplified using the Q5 High-Fidelity DNA Polymerase (NEB). The resulting amplicons were assembled with a pBeloBAC11 backbone (NEB, GenBank Accession #: U51113) modified with CMV and T7 promotors and a bGH polyA signal (amplified from pcDNA4, Thermo Fisher Scientific, V86320) as well as the HDV ribozyme (synthesized as gene block by Integrated DNA Technologies IDT) using the NEBuilder HiFi DNA Assembly Cloning Kit (NEB). Assembled DNA was electroporated into the *E. coli* GS1783 strain and the resulting clones designated pBSCoV-2 were confirmed by restriction digestion and next-generation sequencing (MiSeq™, Illumina). The viral ORF6 gene was replaced with EYFP by homologous recombination using the two-step Lambda-Red Recombination System [13]. Positive clones of pBSCoV2 d6-YFP were confirmed by restriction digestion and next generation sequencing. For virus reconstitution, a co-culture of HEK293T cells stably expressing either ACE2 (ref [30] cloned into pLV-EF1a-IRES-Blast, Addgene #85133) or the viral N protein (amplified from patient material; cloned into pLV-EF1a-Blast) and T7-RNA polymerase (amplified from pCAGT7, kindly provided by Marco Thomas, Virology, FAU, Erlangen; cloned into pLV-EF1a-IRES-Puro, Addgene #85132) was transfected with pBSCoV2 d6-YFP using GenJet™ Reagent (II) (SignaGen^®^) according to the manufacturer’s protocol. At three d p.t., the supernatant was transferred onto Caco-2 cells for passage 1 (P1) virus stocks. Passage 2 (P2) virus stocks were obtained after infection of Caco-2 cells with 1:50 dilution of P1 virus. Viral titers of rec-SARS-CoV-2 d6-YFP were determined by endpoint titration on Caco-2 cells.

### 4.3. Plaque Formation Assay

Caco-2 cells were seeded in 6-well plates to grow confluent monolayers and were used for the infection with serial dilutions of the d6-YFP reporter virus. At two h p.i., the inoculum was removed and replaced with medium containing 1.5% Avicel RL-591 (IFF Nutrition & Biosciences, Oegstgeest, The Netherlands). Cells were incubated for 3 days and fixed by the addition of an equal volume of 8% PFA in PBS for at least 2 h at 4 °C. Afterwards, the overlay was carefully removed, cells were washed at least three times with PBS to completely remove the overlay medium, before fluorescent virus plaques were imaged with an Advanced Fluorescence Imager (Intas Science Imaging, Göttingen, Germany).

### 4.4. Antiviral Compounds

RDV (Gilead, Foster City, CA, USA) and GC376 (TargetMol, Boston, MA, USA) were used as reference compounds to assess the anti-SARS-CoV-2 in vitro activity or viral 3CL^pro^ protease activity, respectively. The CDK inhibitors LDC4297 (Lead Discovery Center, GmbH, Dortmund, Germany), SNS 032 (Tocris, Bristol, United Kingdom) and R25 (also termed alsterpaullone, GPC Biotech AG, Martinsried, Germany) were obtained from indicated sources.

### 4.5. RT-qPCR for the Detection of Extracellular SARS-CoV-2

For measurements with virus-specific RT-qPCR, inactivated viral supernatants were digested with proteinase K (final concentration of 0.136 mg/mL) for 1 h at 56 °C followed by 5 min heat inactivation at 95 °C and a dilution of 1:10 in H_2_O. For further analysis, volumes of 5 µL of the digested supernatants were used and the RT-qPCR was performed according to AgPath-ID™ One-Step RT-PCR (AM1005, Thermo Fisher Scientific) or NEB Luna Universal Probe One-Step RT-qPCR (E3006, NEB). Primer sequences were adapted from Corman et al., 2020 ([31], RdRp_SARSr-F and RdRp_SARSr-R). The probe (caggtggaacctcatcaggagatgc) was 5′ labeled with 6-FAM (6-carboxyfluorescein) and 3′ with BHQ-1 (Black Hole Quencher 1). All oligonucleotides were purchased from Biomers.net (Ulm, Germany).

### 4.6. In-Cell Immunostaining for the Detection of Intracellular SARS-CoV-2

In-cell immunostaining for assessment of SARS-CoV-2 replication was performed similar to previously described experimental approaches [24] by implementing fluorescence labels to facilitate the highly quantitative detection of different antigens as well as microscopic imaging of infected cells. Formalin-fixed cells were permeabilized with 0.2% Triton X-100 in PBS and blocked with blocking buffer (1% BSA in PBS). Cells were sequentially incubated with primary and secondary antibodies diluted in blocking buffer, with two PBS washing steps after each antibody incubation. A new mouse monoclonal against the viral spike (S) protein (mAb-S TRES-6.18) was produced as described previously and used for staining the viral S protein (in-cell immunofluorescence protein-specific assay) [24]. Double-stranded RNA (in-cell immunofluorescence dsRNA-specific assay) was detected by the mAb J2 (SCIONS) [32] specifically recognizing RNA helices of at least 40 bp while being inert towards other nucleic acids present in uninfected cells. Alexa 647- or Alexa 488-labeled goat anti-mouse secondary antibodies were used for quantitative detection or microscopic imaging, respectively (anti-mouse Alexa 647, A-21236, ThermoFisher Scientific; anti-mouse Alexa 488, A11029, ThermoFisher Scientific). For quantitative detection of Alexa 488 in the context of cells harboring YFP fluorescence, bound antibodies were eluted by incubating the antibody-stained cells with 62.5 mm Tris pH 6.8, 100 mm β-mecaptoethanol and 2% SDS at 37 °C for 2 h [33]. Fluorescence was determined to after the transfer of the eluate in a new multiwell plate by a Victor X4 multilabel reader (Perkin Elmer) to allow detection of Alexa 488 in the absence of YFP. Cell layers were used for subsequent restaining procedures after several washing steps with PBS. For microscopic analyses of cells displaying endogenous YFP fluorescence, viral antigens were stained with an appropriate primary antibody followed by an Alexa 647-conjugated goat secondary antibody and imaged by an ImmunoSpot^®^ S6 ULTIMATE UV Image Analyzer (Cellular Technology Limited/CTL, Cleveland, OH, USA). Parallel to antibody staining, one of the two fluorescent DNA dyes SYTOX Blue or Hoechst 33342 (both ThermoFisher) was used as an internal control for estimating cell counts in Victor X4 or the ImmunoSpot reader measurements, respectively.

### 4.7. Two Plasmid-Based Approaches to Analyze SARS-CoV-2-Specific Protease Inhibitors: The New FRET CFP::YFP-Based 3CL^pro^ Activity Assay (FRET Assay) and the Recently Established FlipGFP-Based 3CL^pro^ Activity Assay (FlipGFP Assay)

The FlipGFP and 3CL^pro^ expression plasmids were kindly provided by Nicholas Heaton, Duke University Molecular Genetics and Microbiology, Durham, NC, USA. Expression plasmids coding for FRET CFP::YFP and FRET CFP::YFP T2A were generated by standard polymerase chain reaction (PCR) amplification. Oligonucleotide primers used for PCR were purchased from Biomers.net (Table S1, Ulm, Germany). After cleavage with the corresponding restriction enzymes, PCR products were inserted into the Flip-GFP backbone (pLEX). Both assays were performed under very similar experimental conditions. HEK 293T cells were cultivated at 37 °C, with 5% CO_2_ and 80% humidity using Dulbecco’s modified Eagle’s medium (DMEM, 11960044, Thermo Fisher Scientific) supplemented with 1× GlutaMAX™(35050038, Thermo Fisher Scientific), 10 µg/mL gentamicin (22185.03, SERVA) and 10% fetal bovine serum (FBS, F7524, Sigma Aldrich). Cells were seeded in a 10 cm dish at about 80% confluency and transient cotransfection of the FlipGFP or FRET CFP::YFP with 3CL^pro^ was performed with polyethylenimine-DNA complexes (Sigma-Aldrich) as described previously [34]. At 4 h p.t., cells were trypsinized and transferred onto a 96-well plate and immediately treated with GC376 or RDV at indicated concentrations. At 19 h p.t., cells were analyzed by a Victor X4 microplate reader (PerkinElmer, Waltham, MA, USA). Subsequently, a Neutral Red assay was performed as described previously [24] to determine the cytotoxicity. Western blot analysis was performed by standard procedures as described previously [35], using mAb-GFP (11814460001, Roche) and mAb-β-Actin (A5441, Sigma Aldrich, St. Louis, MO, USA) as primary staining antibodies. For FRET measurements, three fluorescent parameters were determined in parallel by using appropriate combinations of light sources and detection filters (excitation/emission given in nm) for CFP (436/480), YFP (485/353) and FRET (436/353). In the uncleaved FRET CFP::YFP construct, the excitation of CFP leads to an energy transfer to YFP and thus to an emission of YFP signal. In the cleaved construct, however, the so-called FRET signal energy transfer is disrupted in a way that no YFP is emitted any longer through the excitation of CFP. The obtained raw fluorescence values were background corrected by using untransfected cells. Resulting FRET values were normalized for the amount of total CFP and YFP fluorescence to account for potential differences in cell densities. The FRET signal for single transfected FRET CFP::YFP served as the 100% reference (no cleavage, maximal FRET). The mock-treated cotransfection of FRET CFP::YFP with 3CL^pro^ was set to 0% (maximal cleavage, lowest observable FRET). This step also corrects the FRET signal for donor and acceptor spectral bleed through, and thus makes the parallel measurement of CFP and YFP single transfections redundant since all FRET CFP::YFP-containing samples are expected to yield comparable amounts of total CFP and YFP fluorescence.

### 4.8. Drug Combination Loewe Fixed-Dose Assay Adapted to SARS-CoV-2 d6-YFP Infection

Loewe additivity was assessed using an adapted protocol of the HCMV GFP-based replication assay described previously [18]. Infection of Caco-2 cells was performed as described above. Concentrations of antiviral test compounds were added as twofold serial dilutions starting at 4 × EC_50_ as highest concentration and comprising seven dilution steps of each single compound and well as the combination thereof. All infections were performed in biological quadruplicates. Viral replication was assessed by YFP quantitation in a Victor X4 microplate reader (PerkinElmer, Waltham, MA, USA) as described above. Antiviral efficacy (mean of biological quadruplicates) was expressed as the percentage of mock-treated control and entered into the CompuSyn software (Version 1.0; Chou, T.C.; Martin, N. 2005, CompuSyn for drug combinations; ComboSyn, Inc., Paramus, NJ, USA). CI values at 50, 75, 90 and 95% virus inhibition were used for final evaluation.

## Data Availability

Data is contained within the article and Supplementary Material.

## Supplementary Materials

The following are available online at https://www.mdpi.com/article/10.3390/pathogens10091076/s1. Table S1. Oligonucleotide primers used in this study. Figure S1: Steps of optimization of the FlipGFP assay: protease/reporter ratios.
