# Supplementary material for: Methodological Development of a Multi-Readout Assay for the Assessment of Antiviral Drugs against SARS-CoV-2
Source: Pathogens. 2021 Aug 25;10(9):1076. doi: 10.3390/pathogens10091076 (PMC8466411; doi:10.3390/pathogens10091076)
Supplement: Supplementary file 1 [file pathogens-10-01076-s001.zip › pathogens-1300421-supplementary.pdf]

Supplementary Materials

Methodological development of a multi-readout assay for the assessment of antiviral drugs against SARS-CoV-2

Friedrich Hahn [1], Sigrun Häge [1], Alexandra Herrmann [1], Christina Wangen [1], Jintawee Kicuntod [1], Doris Jungnickl [1], Julia Tillmanns [1], Regina Müller [1], Kirsten Fraedrich [1], Klaus Überla [1], Hella Kohlhof [2], Armin Ensser [1] and Manfred Marschall [1,*]

1 Institute for Clinical and Molecular Virology, Friedrich-Alexander University of Erlangen-Nürnberg (FAU), Erlangen, Germany; friedrich.hahn@uk-erlangen.de (F.H.); sigrun.haege@fau.de (S.H.); alexandra.herrmann@uk-erlangen.de (A.H.); christina.wangen@uk-erlangen.de (C.W.); jintawee.kicuntod@extern.uk-erlangen.de (J.K.); doris.jungnickl@uk-erlangen.de (D.J.); jul.tillmanns@fau.de (J.T.); mueller.regina@uk-erlangen.de (R.M.); kirsten.fraedrich@uk-erlangen.de (K.F.); klaus.ueberla@fau.de (K.Ü.); armin.ensser@fau.de (A.E.); manfred.marschall@fau.de (M.M.)

2 Immunic AG, Gräfelfing, Germany; hella.kohlhof@imux.com (H.K.)

* Correspondence: manfred.marschall@fau.de; phone +49 9131 8526089

Table S1. Oligonucleotide primers used in this study. The following information is given by the sequence description: translational start or stop codons (capital letters, underlined), restriction sites (capital letters, bold), additional bases (lower case letters) and coding sequences (capital letters).

Primer	Sequence (5' → 3')
5-BamHI-eCFP	tag**GGATCC**<u>ATG</u>GTGAGCAAGGGCGAGGAG
3-XbaI-NotI-eYFP	tag**TCTAGA**ctcga**GCGGCCGC**<u>TTA</u>CTTGTACAGCTCGTCCATG
5-3CL pro Cleavage-SpeI-eYFP	GC**GCTAGC**GTGGCCAGACTGCAGAGCGGCTTC**ACTAGT**GGCAGCGTGAGCAAGGGCGAGGAG
3-3CL pro Cleavage-NheI-eCFP	CCACTAGTGAAGCCGCTCTGCAGTCTGGCCAC**GCTAGC**GCTGCCCTTGTACAGCTCGTCCATGC
5-T2A Cleavage-eYFP	GGGCAGCCTGCTGACCTGCGGCGACGTGGAGGAGAACCCCGGCCCCGTGAGCAAGGGCGAGGAG
3-T2A Cleavage-eCFP	GGGGTTCTCCTCCACGTCGCCGCAGGTCAGCAGGCTGCCCCGGCCCTCGCCGCTGCCCTTGTACAGCTCGTCCATG

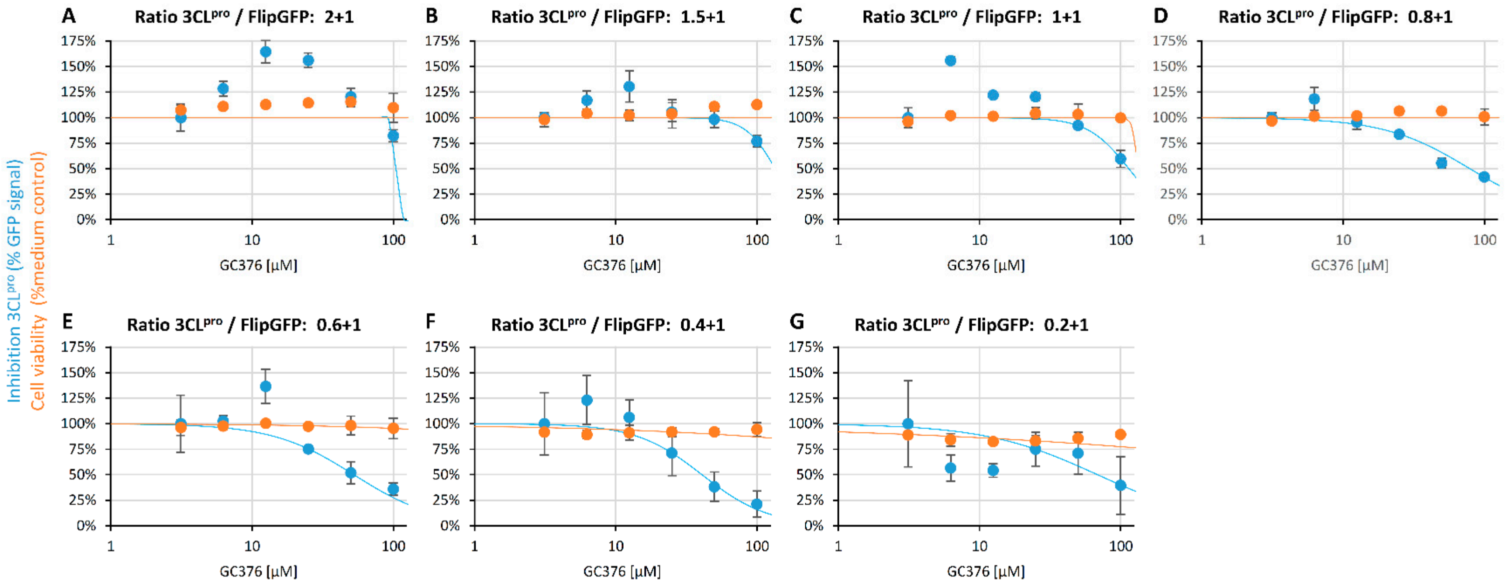

Figure S1: Steps of optimization of the FlipGFP assay: protease/reporter ratios **(A-G)**. Several different ratios of transfected plasmids coding for the protease or reporter protein, respectively, were applied as indicated.
